# Construction of Heterostructured Ni_3_S_2_@V-NiFe(III) LDH for Enhanced OER Performance

**DOI:** 10.3390/molecules29246018

**Published:** 2024-12-20

**Authors:** Qianqian Dong, Qijun Zhong, Jie Zhou, Yuhao Li, Yujing Wang, Jiayang Cai, Shuangwei Yu, Xiong He, Shaohui Zhang

**Affiliations:** 1 Liuzhou Key Laboratory of New Energy Vehicle Power Lithium Battery, Guangxi Engineering Research Center for Characteristic Metallic Powder Materials, School of Electronic Engineering, Guangxi University of Science and Technology, Liuzhou 545000, China; isdongqq@163.com (Q.D.); zqj17878906682@163.com (Q.Z.); 17586600924@163.com (J.Z.); q2476166290@126.com (Y.L.); wang_yu_jing01@163.com (Y.W.); yushuangwei2023@163.com (S.Y.); zhangshaohui@gxust.edu.cn (S.Z.); 2 Guangxi Key Laboratory of Green Processing of Sugar Resources, College of Biological and Chemical Engineering, Guangxi University of Science and Technology, Liuzhou 545006, China; cjy193677464@163.com

**Keywords:** heterostructure, electronic structure regulation, V-doping, layered double hydroxide, oxygen evolution reaction

## Abstract

The oxygen evolution reaction (OER), which involves a four-electron transfer and slow kinetics, requires an efficient catalyst to overcome the high energy barrier for high-performance water electrolysis. In this paper, a novel Ni_3_S_2_@V-NiFe(III) LDH/NF catalyst was prepared via a facile two-step hydrothermal method. The constructed heterostructure of Ni_3_S_2_@V-NiFe(III) LDH increases the specific surface area and regulates the electronic structure. Furthermore, the introduction of the V element forms an electron transport chain of Ni-O-Fe-O-V-O-Ni, which optimizes the binding energy between metal active sites and oxygen evolution reaction intermediates, accelerates electron transfer, and improves self-reconstruction. With this dual regulation strategy, Ni_3_S_2_@V-NiFe(III) LDH/NF exhibits exceptional OER performance with an overpotential of 280 mV at 100 mA/cm^2^ and a Tafel slope of 45.4 mV/dec. This work develops a dual regulation strategy combining heterostructure formation and the doping effect, which are beneficial in the design of efficient OER catalysts.

## 1. Introduction

The growing energy demand and intensifying environmental crisis have positioned hydrogen as a promising green energy source with high energy density and zero carbon emission [1,2]. Water electrolysis, which involves an anodic oxygen evolution reaction (OER) and a cathodic hydrogen evolution reaction (HER), is an important way to produce green and high-purity hydrogen on a large scale. During water electrolysis, OER is considered as the rate-limiting step due to slow four-electron transfer kinetics [3,4]. Despite the fact that noble metal oxides (IrO_2_/RuO_2_) display excellent OER catalytic activity, their rarity and elevated cost restrict their large-scale application. Therefore, it is a significant challenge to develop efficient, cost-effective, and durable non-noble metal-based catalysts [5,6,7].

In recent years, transition metal-based catalysts, particularly those incorporating Fe, Co, and Ni, have garnered significant attention owing to their favorable OER activity in alkaline electrolytes [8]. This favorable activity is attributed to their inherent alkaline resistance and rich electronic structure. In particular, nickel–iron layered double hydroxides (NiFe LDHs) have emerged as highly efficient OER electrocatalysts with tunable compositions and an adjustable crystal structure [9,10,11,12]. The unique sandwich-like structure of LDHs, consisting of positively charged octahedral layers [M^2+^_1−x_M^3+^_x_(OH)_2_]_x_^+^ and interlayers filled with anions and water molecules, plays an important role in their catalytic activity [13]. Nevertheless, NiFe LDHs still face challenges, such as poor electrical conductivity, limited surface active sites, and inadequate stability, which hinder their practical application [14]. As the electrochemical performance of catalysts is tightly associated with their electronic structure, tailoring the electronic structure is crucial for the development and design of efficient electrocatalysts.

Heterostructure engineering has emerged as an effective strategy to boost OER activity by improving interfaces and tuning electronic structures. It is believed that the interfaces within the heterostructure between different components are believed to be the real catalytic active sites [15,16]. The selection of materials for constructing heterojunctions is critical for achieving a promising performance. A variety of heterostructured OER electrocatalysts have been explored based on metal sulfides, selenides, nitrides, carbides, and phosphides [17]. Notably, Ni_3_S_2_ has garnered attention due to its low cost and intrinsic metallic behavior, attributed to the continuous network of Ni−Ni bonds [18,19]. Additionally, Ni_3_S_2_ serves as an excellent conductive substrate, facilitating tight connections with active materials [20,21]. Consequently, Ni_3_S_2_-based heterostructured catalysts have been widely studied in OER applications because of their unique structural configuration and high electrical conductivity [22]. For instance, Luo et al. successfully constructed tree arrays of NiS/Ni_3_S_2_ heterostructures on nickel foam substrates using a one-step hydrothermal method. The NiS-Ni_3_S_2_ heterostructure arrays exhibited a synergistic effect, improving OER performance with an overpotential of 269 mV to achieve a current density of 10 mA/cm^2^ [23]. Zhang et al. prepared a Ni_3_S_2_-Co_9_S_8_/NF heterostructured nanowire catalyst via a two-step hydrothermal method. The catalyst exhibited excellent OER activity with an overpotential of only 294 mV at 20 mA/cm^2^, likely due to defects at the heterogeneous interface between the metallic Co_9_S_8_ and Ni_3_S_2_ nanograins [24]. Similarly, Lin et al. developed a Ni_3_Se_2_/Ni_3_S_2_/NF catalyst with a heterogeneous structure, achieving excellent OER performance with low overpotentials of 251 and 312 mV at 50 and 100 mA/cm^2^, respectively [25]. The structures and properties of other materials in the heterostructure are also crucial. Most of the reported heterojunction structures still suffer from shortcomings such as sparse interfacial sites and complex preparation processes [15]. Selecting suitable materials and schemes remains challenging. Yet fewer studies have combined heterojunction construction with other strategies to improve catalyst performance through synergistic effects.

Doping high-valence transition metals, such as tantalum [26], tungsten [27], cobalt [28], and vanadium, into catalysts is another promising approach to enhance OER activity [29]. Such high-valence cation-doping can induce electron transfer from Fe or Ni to a high-valence metal, thus optimizing the electronic structure for an OER [26]. Vanadium (V) has proven to be a particularly effective dopant, enhancing catalytic sites, improving intrinsic catalytic activity, and stabilizing charge transfer [30,31,32]. For example, Kong et al. prepared a NiFe-V_1.0_ catalyst by a one-step hydrothermal method, which reduced the oxygen evolution overpotential by 37 mV at a current density of 10 mA/cm^2^ and decreased the Tafel slope by 43.37 mV/dec compared to undoped NiFe LDH [33]. Zhou et al. developed V-Ni_3_S_2_@NiFe LDHs with the required overpotentials to reach current densities of 10 and 100 mA/cm^2^, 209 and 286 mV, respectively [34]. Jiang et al. reported the incorporation of Fe and V into nickel hydroxide lattices resulted in the formation of a Ni-O-Fe-O-V-O-Ni electron transport chain, significantly enhancing OER activity, by modulating the local coordination environment and electronic structure of cations [30]. Despite these advances, the combination of heterostructure and doping strategy is still challenging in designing efficient OER catalysts.

Inspired by this knowledge, we employ a dual regulation strategy that utilizes the synergistic effects of Ni_3_S_2_@NiFe LDH heterostructure formation and vanadium-doping to enhance OER performance. A Ni_3_S_2_@V-NiFe(III) LDH/NF with interleaved ultra-thin nanosheets has been synthesized by a facile two-step hydrothermal method. The as-prepared Ni_3_S_2_@V-NiFe(III) LDH/NF exhibits optimal OER performance due to dual regulation of its electronic structure. It possesses a low overpotential of 280 mV at a current density of 100 mA/cm^2^ and excellent stability at 50 mA/cm^2^ up to 100 h in a 1 M KOH alkaline electrolyte. SEM and TEM analyses show that Ni_3_S_2_ composite V-NiFe LDH forms a heterostructure of interlaced ultra-thin and ultra-large nanosheets that specifically enhances the surface area and conductivity of the material. Shifts of binding energies in the XPS spectra reveal electron transfer within the Ni-O-Fe-O-V-O-Ni. Raman spectra, combined with CV analysis, demonstrates the Ni-O-Fe-O-V-O-Ni electron transport chain not only promotes charge transfer, but also accelerates the formation of active sites and improves self-reconstruction.

## 2. Results and Discussion

### 2.1. Morphology and Structure Characterization

**Figure 1:**
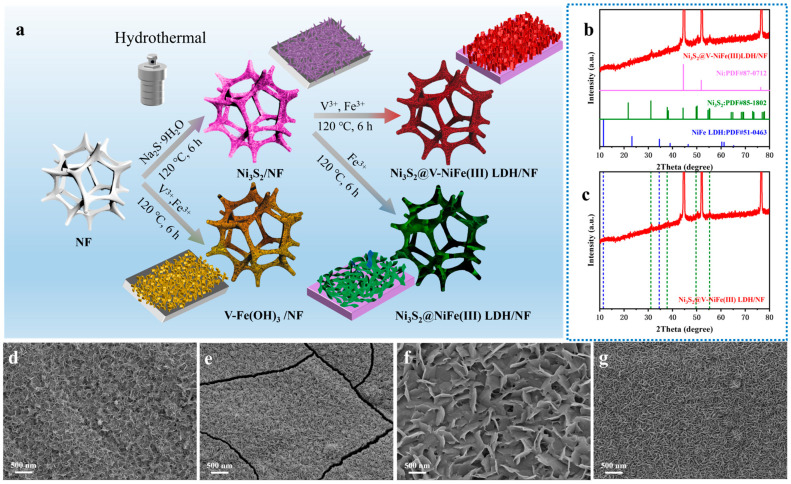
(**a**) A schematic illustration of the formation processes of Fe(III)-series electrocatalysts, (**b**,**c**) XRD patterns of Ni_3_S_2_@V-NiFe(III) LDH/NF, and SEM images of (**d**) Ni_3_S_2_/NF, (**e**) V-Fe(OH)_3_/NF, (**f**) Ni_3_S_2_@NiFe(III) LDH/NF, and (**g**) Ni_3_S_2_@V-NiFe(III) LDH/NF.

Figure 1a illustrates the synthesis of Fe(III)-series electrocatalysts (Ni_3_S_2_@V-NiFe(III) LDH/NF, V-Fe(OH)_3_/NF, Ni_3_S_2_@NiFe(III) LDH/NF, and Ni_3_S_2_/NF) using a simple one- or two-step hydrothermal method. The composition and crystal structure of Ni_3_S_2_@V-NiFe(III) LDH/NF were determined by XRD, as shown in Figure 1b. Strong diffraction peaks at 44.6°, 51.9°, and 76.5° originate from the base NF(PDF#87-0712). The diffraction peaks at 31.1°, 37.8°, 49.7°, and 55.3° correspond to the (−110), (111), (210), and (121) planes of Ni_3_S_2_ (PDF# 85-1802), respectively. The diffraction peaks at 11.5° and 34.6° are assigned to the (003) and (012) planes of NiFe LDH (PDF# 51-0463). Additionally, other samples were determined by XRD testing and the results are shown in Figure S1. The diffraction peaks of N_3_S_2_, represented by a five-pointed star, were detected in N_3_S_2_/NF, Ni_3_S_2_@NiFe(III) LDH/NF, and Ni_3_S_2_@V-NiFe(III) LDH/NF. The diffraction peaks of NiFe LDH, represented by a triangle, were detected in Ni_3_S_2_@NiFe(III) LDH/NF and Ni_3_S_2_@V-NiFe(III) LDH/NF. There were no diffraction peaks of NiFe LDH detected in V-Fe(OH)_3_. The diamond shape represents the diffraction peaks of V_0.875_Fe_0.125_O(OH) (PDF#73-0513). This indicates the formation of FeV hydroxide.

The morphology and structure of Fe(III)-series electrocatalysts were observed by SEM, as illustrated in Figure 1d–g. Ni_3_S_2_ nanosheets grew directly on the NF (Figure 1d), forming a continuous network which facilitated subsequent in situ growth of NiFe LDH. As Figure 1e shows, V-Fe(OH)_3_/NF is composed of short and thick interlaced nanosheets. NiFe(III) LDH formed a heterostructure on Ni_3_S_2_, enlarging its wave-like flakes (Figure 1f). The Ni_3_S_2_@V-NiFe(III) LDH/NF structure (Figure 1g) features thinner, larger nanosheets growing vertically on the Ni_3_S_2_ substrate, resulting in enhanced support and a well-defined 3D structure. The dual-regulation-combined Ni_3_S_2_ composite and V-doping granted Ni_3_S_2_@V-NiFe(III) LDH/NF a larger specific surface area, increased porosity, stronger structural support, and improved stability. As a result, this structure exposed more active sites and facilitated faster oxygen evolution reaction (OER) kinetics by allowing for a more efficient release of oxygen.

**Figure 2:**
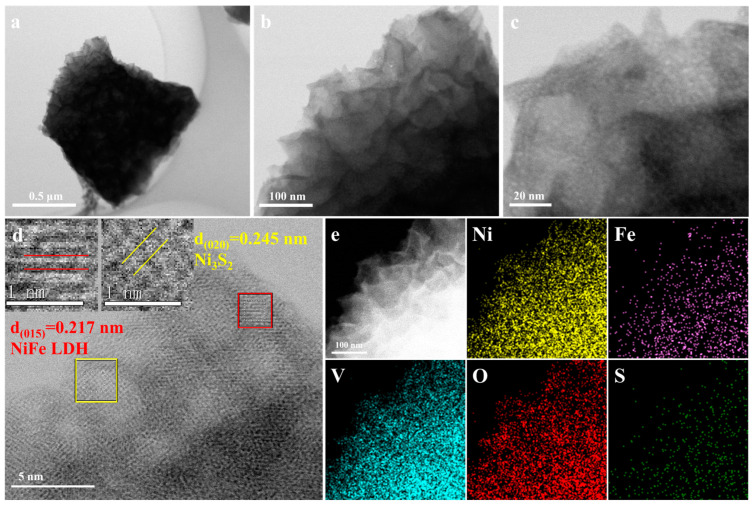
(**a**–**c**) TEM images of Ni_3_S_2_@V-NiFe(III) LDH/NF, (**d**) HRTEM image of Ni_3_S_2_@V-NiFe(III) LDH/NF, and (**e**) EDX elemental mapping images of Ni_3_S_2_@V-NiFe(III) LDH/NF.

TEM was used to characterize the crystal structure and morphology of the Ni_3_S_2_@V-NiFe(III) LDH/NF sample, as shown in Figure 2. The nanosheet structure can be obtained with Ni_3_S_2_ as the bottom layer and V-NiFe(III) LDH as the outer layer, as shown in Figure 2a–c, consistent with the SEM results. A HRTEM image (Figure 2d) demonstrates distinct interplanar spacings of 0.245 and 0.217 nm corresponding to the (020) plane of Ni_3_S_2_ (PDF#85-1802) and (015) plane of NiFe LDH (PDF #51-0463), respectively. This result reveals the formation of a heterostructure between Ni_3_S_2_ and V-NiFe(III) LDH. Figure 2e presents EDX elemental mapping in a select region of Ni_3_S_2_@V-NiFe(III) LDH/NF. It can be seen that Ni, Fe, V, O, and S are uniformly distributed in the electrocatalyst. The S element is primarily distributed in the inner layer, which further proves the hierarchical heterojunctive structure of V-NiFe LDH nanosheets covered on Ni_3_S_2_ flakes.

**Figure 3:**
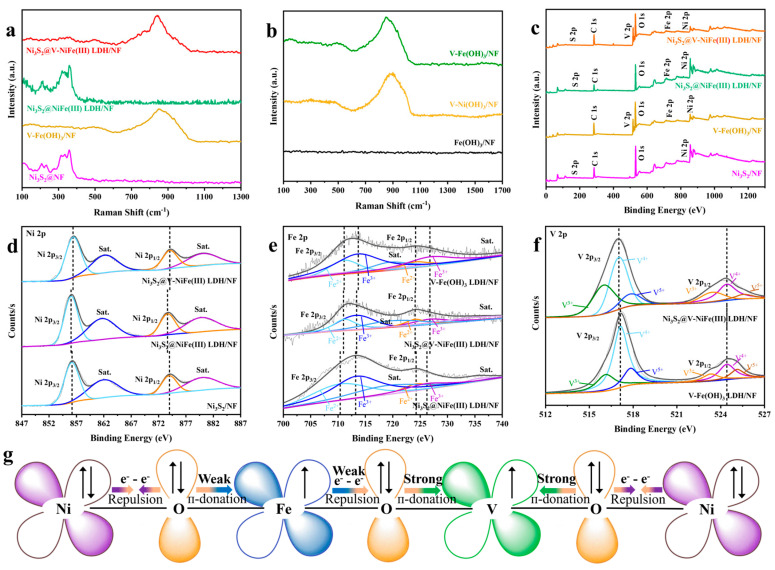
Raman spectra of Fe(III)-series samples: (**a**) Ni_3_S_2_@V-NiFe(III) LDH/NF, Ni_3_S_2_@NiFe(III) LDH/NF, V-Fe(OH)_3_/NF, and Ni_3_S_2_/NF, and (**b**) V-Fe(OH)_3_/NF, V-Ni(OH)_3_/NF, and Fe(OH)_3_/NF. XPS spectra of Fe-series samples: (**c**) XPS survey spectra, (**d**) Ni 2p of Ni_3_S_2_@V-NiFe LDH(III)/NF, Ni_3_S_2_@NiFe(III) LDH/NF, and Ni_3_S_2_/NF, (**e**) Fe 2p of V-Fe(OH)_3_/NF, Ni_3_S_2_@V-NiFe(III) LDH/NF, and Ni_3_S_2_@NiFe(III) LDH/NF, and (**f**) V 2p of Ni_3_S_2_@V-NiFe(III) LDH/NF and V-Fe(OH)_3_/NF. (**g**) Schematic representation of Ni-O-Fe-O-V-O-Ni electronic coupling.

Raman spectra (Figure 3a) reveal the heterogeneous structure and composition of these samples. Peaks at 197, 219, 306, and 347 cm^−1^ can be observed in Ni_3_S_2_/NF, Ni_3_S_2_@NiFe(III) LDH/NF, and Ni_3_S_2_@V-NiFe(III) LDH/NF, which correspond to the polarization modes of Ni_3_S_2_ [19]. The peak at 486 cm^−1^, attributed to the Eg bending vibration mode of M-O bonds, increases significantly with V-doping, indicating enhanced V-O-M interaction [35]. Peaks around 400–500 and 600–1000 cm^−1^ are associated with V-O vibrations, further confirming the formation of V-O-M bonds, which are crucial for OER performance [36]. Similarly, the Raman spectra of the Ni_3_S_2_@V-NiFe(III) LDH/NF and V-Fe(OH)_3_/NF samples show wide peaks ranging from 600 to 1000 cm^−1^, attributed to the interaction among the Eg rotation patterns of Ni-OH (877 cm^−1^), Fe-O (680 cm^−1^), and V-O (600–1000 cm^−1^) [21]. The Raman spectra detected Fe(OH)_3_/NF, V-Ni(OH)_3_/NF, and V-Fe(OH)_3_/NF and served to verify the effect of V-doping, as shown in Figure 3b. V-Ni(OH)_3_/NF and V-Fe(OH)_3_/NF possess similar characteristic peaks located at 486 and 600~1000 cm^−1^. No obvious characteristic peaks were detected for Fe(OH)_3_/NF. This phenomenon further indicates that the wide peaks at 486 and 600~1000 cm^−1^ are caused by V-O-M bonds rather than other factors such as iron oxides. It also suggests the successful V-doping in Ni_3_S_2_@V-NiFe(III) LDH/NF.

The surface valence state and chemical composition of these catalysts were determined by XPS. The XPS survey spectra of Ni_3_S_2_/NF, V-Fe(OH)_3_/NF, Ni_3_S_2_@NiFe(III) LDH/NF, and Ni_3_S_2_@V-NiFe(III) LDH/NF samples are presented in Figure 3c. They confirm successful V-doping in Ni_3_S_2_@V-NiFe(III) LDH/NF and V-Fe(OH)_3_/NF. Figure 3d demonstrates the Ni 2p XPS spectra of Ni_3_S_2_/NF, Ni_3_S_2_@NiFe(III) LDH/NF, and Ni_3_S_2_@V-NiFe(III) LDH/NF. Ni 2p_3/2_ and 2p_1/2_ peaks, located at 856.2/856.1 and 874.1/873.9 eV, can be observed for Ni_3_S_2_/NF and Ni_3_S_2_@NiFe(III) LDH/NF, respectively. The slight deviations are attributed to the amorphous nature of the Ni_3_S_2_ nanosheets, which can induce a large number of vacancies resulting in a higher oxidation state of Ni atoms [21]. Compared to Ni_3_S_2_@NiFe(III) LDH/NF, Ni_3_S_2_@V-NiFe(III) LDH/NF demonstrates a positive shift in the Ni 2p_3/2_ and 2p_1/2_ peaks (856.3 and 874.1 eV). The positive shifts illustrate that the electronic structure can be changed after V-doping, evidenced by the loss of electrons in the Ni atoms of Ni_3_S_2_@V-NiFe(III) LDH/NF.

Figure 3f exhibits the Fe 2p XPS spectra of V-Fe(OH)_3_/NF, Ni_3_S_2_@NiFe(III) LDH/NF, and Ni_3_S_2_@V-NiFe(III) LDH/NF. For Ni_3_S_2_@NiFe(III) LDH/NF, two pairs of peaks at 710.3/712.8 and 724.1/725.8 eV were observed, which are associated with Fe 2p_3/2_ and Fe 2p_1/2_ of Fe^2+^ and Fe^3+^, respectively. After V-doping, the peaks at 711.1/713.5 and 724.3/727.2 eV were observed, corresponding to Fe 2p_3/2_ and Fe 2p_1/2_ of Fe^2+^ and Fe^3+^, respectively [30]. These positive shifts are consistent with the binding energy change of Ni in Ni_3_S_2_@V-NiFe(III) LDH/NF and are attributed to V-doping by inducing a transfer of electrons from Ni and Fe to V and forming the electron-transfer mechanism for M-O-V. V^4+^ and V^5+^ have empty e_g_ orbitals that form a strong bond with absorbed oxygen, while Ni^2+^ and Fe^3+^ have half-full e_g_ orbitals, leading to a weaker bond with oxygen. According to the Sabatier principle, the moderate bonding strength between the transition metal and the absorbed oxygen is more conducive to the catalytic OER process [26,37,38].

The V 2p XPS spectra of Ni_3_S_2_@V-NiFe(III) LDH/NF and V-Fe(OH)_3_/NF are demonstrated in Figure 3f. The V 2p_3/2_ peak can be deconvoluted into three peaks at 515.9 eV (V^3+^), 517.1 eV (V^4+^), and 517.7 eV (V^5+^) [30]. Similarly, the V 2p_1/2_ peaks can be divided into three peaks at 523.5 eV (V^3+^), 524.5 eV (V^4+^), and 525.3 eV (V^5+^). It can be observed that V^4+^ acts as the dominant of the V valence state, indicating the oxidation of V^3+^ to a higher valence during the hydrothermal process [20,31,39].

### 2.2. Electrochemical Performance

**Figure 4:**
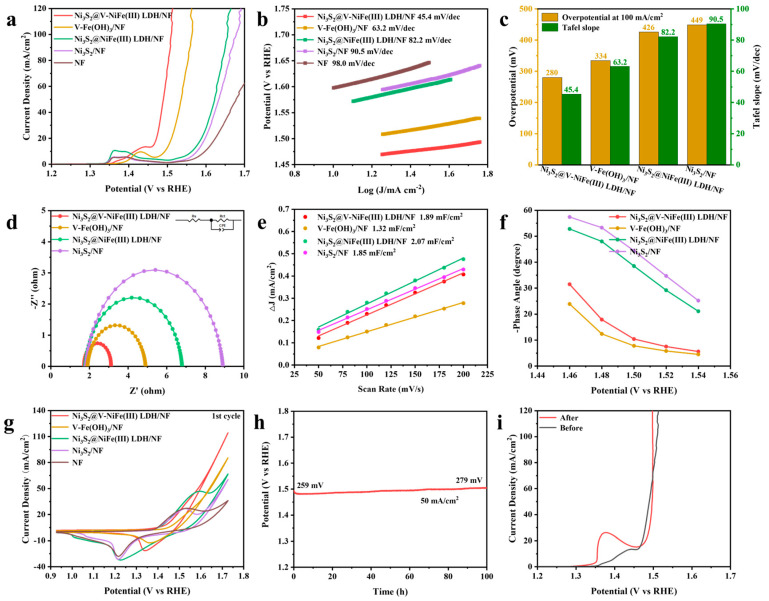
The OER performance of electrocatalysts in a 1 M KOH solution. (**a**) The OER polarization curves with *iR* correction, (**b**) Tafel plots, (**c**) a comparison of the overpotentials required to achieve current densities of 100 mA/cm^2^ and Tafel plots, (**d**) electrochemical impedance spectroscopy (EIS), (**e**) OER double-layer capacitance (C_dl_), (**f**) potential dependence of the phase angle of samples, (**g**) first-cycle CV curves without *iR* correction, (**h**) chronopotentiometry (CP) curve of Ni_3_S_2_@V-NiFe(III) LDH/NF at a constant current density of 50 mA/cm^2^, and (**i**) a comparison of the LSV polarization curves before and after the stability test.

The polarization curves of the synthesized samples, conducted at a scanning rate of 5 mV/s with *iR* correction, are demonstrated in Figure 4a. Compared to Ni_3_S_2_/NF, both Ni_3_S_2_@NiFe(III) LDH/NF and Ni_3_S_2_@V-NiFe(III) LDH/NF exhibit an enhanced OER performance, which can be attributed to heterostructure formation. Additionally, V-Fe(OH)_3_/NF and Ni_3_S_2_@V-NiFe(III) LDH/NF display an outstanding OER performance, suggesting the positive effect of V-doping. Among them, Ni_3_S_2_@V-NiFe(III) LDH/NF possesses an optimal catalytic performance due to the dual regulation effect of V-doping and heterostructure. The peak at 1.35 V represents oxidation of the active site, indicating the generation of NiOOH [40]. From the LSV curves in Figure 4a, it can be clearly seen that the shape and position of the oxidation peaks of the V-doped catalysts are different from the others. This indicates that V-doping caused the generation of a new active substance, likely VOOH, which had higher OER activity [30]. A Tafel slope (β) was further employed to evaluate the kinetic properties of these catalysts, as shown in Figure 4b. Ni_3_S_2_@V-NiFe(III) LDH/NF exhibits the lowest Tafel slope of 45.4 mV/dec, which is superior to V-Fe(OH)_3_/NF (63.2 mV/dec), Ni_3_S_2_@NiFe(III) LDH/NF (82.2 mV/dec), and Ni_3_S_2_/NF (90.5 mV/dec). These results indicate rapid catalytic kinetics in Ni_3_S_2_@V-NiFe(III) LDH/NF, suggesting the rate-determining step at the end of the multi-electron-transfer reaction [41]. The overpotentials at 100 mA/cm^2^ (*η*_100_) and Tafel slopes are summarized in Figure 4c. Ni_3_S_2_@V-NiFe(III) LDH/NF achieves a current density of 100 mA/cm^2^ at an optimal overpotential of 280 mV. Meanwhile, the OER performance of Ni_3_S_2_@V-NiFe(III) LDH/NF is excellent among the transition metal-based electrocatalysts reported in recent years (Table S1).

Electrochemical impedance spectroscopy (EIS) was employed to investigate charge transfer kinetics, as shown in Figure 4d. The smaller the radius of the Nyquist curve, the lower the electrode impedance, which is conducive to electron transfer [42]. The sequence of changes is consistent with previous results indicating Ni_3_S_2_@V-NiFe(III) LDH/NF has the fastest charge transfer rate during the OER process. Electrochemical active surface area (ECSA), as a critical parameter for evaluating the specific surface area of catalysts, is positively correlated with double-layer capacitance (C_dl_). Figure 4e presents the C_dl_ values derived from the cyclic voltammetry (CV) curves conducted at various scan rates (Figure S2). The C_dl_ values of Ni_3_S_2_/NiFe(III) LDH/NF, Ni_3_S_2_@V-NiFe(III) LDH/NF, V-Fe(OH)_3_/NF, and Ni_3_S_2_/NF are calculated as 2.07, 1.89, 1.32, and 1.85 mF/cm^2^, respectively. These results indicate that the heterostructure can provide more active sites, which promotes OER performance. The ECSA was calculated by dividing the C_dl_ by the specific capacitance value of 40 μF/cm^2^ in 1 M KOH [43]. The ECSA-normalized OER polarization curves reflect the inherent activity of the catalysts (Figure S3) [44]. Figure 4f illustrates the potential dependence of the phase angle derived from the Bode plots of the electrocatalysts within a voltage range of 1.46–1.54 V vs. RHE (Figure S4). The phase angles of the V-doped catalysts exhibit a notable decrease. It is known that a greater number of electrons participate in OERs through V-doping [45]. V-doping improves OER efficiency mainly by modulating V-O-M electron transfer. The combination of heterostructure and V-doping results in electronic structure regulation with exceptional OER performance in Ni_3_S_2_@V-NiFe(III) LDH/NF.

The changes in shape and position of the redox peaks following V-doping indicate the oxidized substances have changed and higher current values can be obtained [46]. Redox peaks in the CV curves typically correlate with the interconversion of M(OH)_x_ and MOOH [35]. As shown in Figure 4g, the reduction peaks of Ni_3_S_2_@V-NiFe(III) LDH/NF, V-Fe(OH)_3_/NF, Ni_3_S_2_@NiFe(III) LDH/NF, Ni_3_S_2_/NF, and NF appear at 1.340, 1.362, 1.227, 1.215, and 1.216 V in the 1st-cycle CV curves, respectively. These results indicate that V-doping leads to the formation of new active sites. The 20th-cycle CV curves are shown in Figure S5. Specific values of the reduction peaks can be found in Table S2. The stability of Ni_3_S_2_@V-NiFe(III) LDH/NF was evaluated using a chronoamperometry (CP) plot at a current density of 50 mA/cm^2^, as shown in Figure 4h. After 100 h of continuous testing, the overpotential increased from 259 mV to 279 mV with an increase of only 20 mV, and the LSV curve after the 100-hour stability test exhibited a slight shift (Figure 4i), indicating good stability and durability.

**Figure 5:**
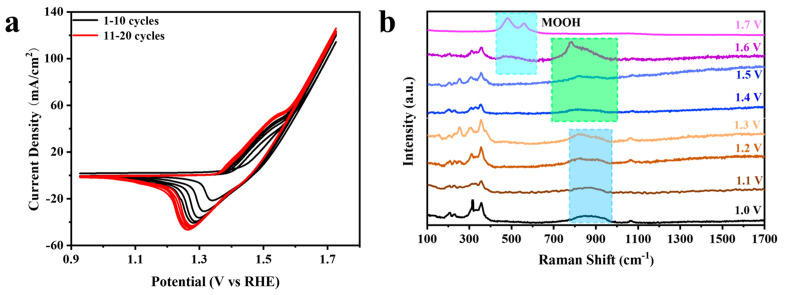
(**a**) The CV curves without *iR* correction and (**b**) Raman spectra acquired at different potentials vs. RHE in the first CV cycle positive scan of Ni_3_S_2_@V-NiFe(III) LDH/NF.

Cyclic voltammetry (CV) was employed to investigate the redox behavior of catalysts under varying potentials [47]. Figure 5a presents the CV curves of Ni_3_S_2_@V-NiFe(III) LDH/NF. During the 20 cycles of CV activation, a significant difference was observed between the first anodic scan and subsequent scans. This result indicates the electrode material is oxidized during the first anodic scan, resulting in the formation of new active material. The area of the redox peaks increases during cycles 1–10 and remains unchanged during cycles 11–20, suggesting that the sample is fully activated [48]. Raman spectroscopy is employed to monitor the anodic scan of the first CV process of Ni_3_S_2_@V-NiFe(III) LDH/NF [49], with the results depicted in Figure 5b. The scan rate of the CV test was 50 mV/s and Raman spectroscopy was performed at 532 nm. The CV test was conducted, in turn, on the material in the electrolytic cell with the voltage set to 0–0.1 V, 0–0.2 V, 0–0.3 V, 0–0.4 V, 0–0.5 V, 0–0.6 V, 0–0.7 V, and 0–0.8 V vs. Hg/HgO. At the conclusion of each test, the electrode material was promptly removed from the electrolytic cell to enable the Raman test to be completed within three minutes, thus ensuring the accuracy of the test. As previously mentioned, peaks at 197, 219, 306, and 347 cm^−1^ correspond to the polarization peaks of Ni_3_S_2_ [19]. The broad peak in the 700–900 cm^−1^ range is attributed to V-O-M-bond interaction. The peak at approximately 140 cm^−1^ is associated with Ni and Fe hydroxide, and the peak at 1066 cm^−1^ indicates CO_3_^2-^ intercalation [50]. The peaks appearing at 477 and 557 cm^−1^ are MOOH [49]. When the voltage rises to 1.6 V, M-OH begins to convert to MOOH, with weakening of the V-O-M interaction and separation of the broad peak. Thus, VOOH at 697.6 cm^−1^ and the Eg rotation pattern of M-OH at 877 cm^−1^ are observed [31]. As the voltage continues to increase, the oxidation process persists, resulting in the formation of additional MOOH-reactive species. At 1.7 V, only MOOH remains. This process aligns with the oxidation peaks in the CV curves.

## 3. Experimental Section

### 3.1. Materials and Chemicals

Nickel foam (NF), with a thickness of 1.0 mm and a purity of 99.9%, was purchased from Taiyuan Lizhiyuan Technology Co., Ltd. (Taiyuan, China). Sodium sulfide (Na_2_S·9H_2_O), vanadium chloride (VCl_3_), urea (CO(NH_2_)_2_), ammonium fluoride (NH_4_F), and ferric sulfate (Fe_2_(SO_4_)_3_) were purchased from Shanghai Aladdin Biochemical Technology Co., Ltd. (Shanghai, China). All the chemicals were of analytical grade and used without further purification.

### 3.2. The Synthesis of the Electrodes

#### 3.2.1. Synthesis of Ni_3_S_2_/NF

The NF was cut into 2 cm × 3 cm and, in turn, put into 1M hydrochloric acid, anhydrous ethanol, and deionized water and ultrasonicated for 15 min for cleaning, after which it was put into a vacuum-drying oven and dried at 60 °C for 6 h.

Ni_3_S_2_/NF was prepared according to the previous report. At first, 3 mmol Na_2_S·9H_2_O was dissolved in 80 mL deionized water. Subsequently, the cleaned NF (2 cm × 3 cm) was taken to a 20 mL Teflon-lined stainless-steel autoclave. Then, 20 mL of the solution was transferred into the reactor, ensuring the NF could be immersed into the solution, and reacted at 120 °C for 6 h. After cooling to room temperature, the resulting product was washed with anhydrous ethanol and deionized water and dried in a vacuum oven at 60 °C for 6 h.

#### 3.2.2. Synthesis of Ni_3_S_2_@V-NiFe(III) LDH/NF and Related Samples

Ni_3_S_2_@V-NiFe(III) LDH/NF was synthesized with the following process: 3 mmol urea, 2 mmol NH_4_F, 0.1 mmol Fe_2_(SO_4_)_3_, and 0.2 mmol VCl_3_ were dissolved in 60 mL deionized water. Subsequently, the previously prepared Ni_3_S_2_/NF was immersed into the solution, transferred into a 20 mL Teflon-lined stainless-steel autoclave, and reacted at 120 °C for 6 h. The cooled product was washed with anhydrous ethanol and deionized water and dried in a vacuum oven at 60 °C for 6 h.

Following a similar procedure as before, V-Fe(OH)_3_/NF was synthesized with NF instead of Ni_3_S_2_/NF. To obtain Ni_3_S_2_@NiFe(III) LDH/NF, the synthesis was carried out without the addition of 0.2 mmol VCl_3_.

### 3.3. Material Characterization

The X-ray diffraction (XRD) patterns of the samples were recorded on a Rigaku SmartLab SE (Neu-Isenburg, Germany) at 40 mA and 40 kV using a monochromatic Cu K*α* radiation (λ = 1.54178Å). Raman spectra were obtained with a Zhuolihanguang/RST2-301-SMS spectrometer (Beijing Zhuo Li Han Kuang Instrument, Beijing, China). Scanning electron microscopy (SEM) measurements were collected with a TESCAN MIRA LMS (TESCAN, Brno, Czechia) operated at 3 kV. Transmission electron microscopy (TEM) and corresponding energy-dispersive X-ray spectroscopy (EDX) analyses were performed with a JEOL/JEM-ARM200F (JEOL, Tokyo, Japan) at 200 KV. X-ray photoelectron spectroscopy (XPS) studies were carried out with a Thermo Scientific K-AIpha (Thermo Fisher Scientific, Waltham, MA, USA).

### 3.4. Electrochemical Measurements

All electrochemical measurements were carried out in a standard three-electrode device using a CHI760E electrochemical workstation (CH Instruments, Inc., Bee Cave, TX, USA) in a 1 M KOH alkaline solution at room temperature. The prepared sample was used as the working electrode (with a test area of 1 cm^2^), the Hg/HgO electrode was used as the reference electrode, and a platinum electrode was used as the counter electrode. Before the electrochemical test, the electrocatalyst was activated by cyclic voltammetry with a scan rate of 50 mV/s for 20 cycles ranging from 0 to 0.8 V vs. Hg/HgO. LSV curves were recorded at a scan rate of 5 mV/s ranging from 0 to 1.0 V vs. Hg/HgO. Electrochemical impedance spectroscopy (EIS) was conducted within a frequency range of 10^5^ to 10^−2^ Hz at open-circuit voltage (OCV). In all electrochemical tests, the potential was adjusted to the reversible hydrogen electrode (RHE) with the formula *E*_RHE_ = *E*_Hg/HgO_ + 0.0592 × pH + 0.098. The overpotential (*η*) was calculated as follows: *η* = *E*_RHE_ − 1.23 V.

## 4. Conclusions

In summary, Ni_3_S_2_@V-NiFe(III) LDH/NF was successfully synthesized by a two-step hydrothermal method. Dual electronic regulation with heterostructure and V-doping was employed to enhance OER performance. Optimal OER performance, with a low overpotential of 280 mV at 100 mA/cm^2^ and a low Tafel slope of 45.4 mV/dec, was achieved in Ni_3_S_2_@V-NiFe(III) LDH/NF. Robust stability is demonstrated with a low overpotential change of 20 mV after a 100-h stability test at 50 mA/cm^2^. This excellent performance can be attributed to its Ni_3_S_2_-based heterostructure, which increases the specific surface area and conductivity. The directed electron transport chain, Ni-O-Fe-O-V-O-Ni, optimizes the binding energy between metal active sites and oxygen evolution reaction intermediates. This unique electron-transfer mechanism also accelerates electron transfer and improves self-reconstruction. Our work offers new ideas in the development of efficient OER catalysts.

## Data Availability

The data presented in this study are available on request from the corresponding author. The data are not publicly available due to privacy.

## Supplementary Materials

The following supporting information can be downloaded at: https://www.mdpi.com/article/10.3390/molecules29246018/s1, Figure S1: XRD patterns of all the samples; Figure S2: Cyclic voltammograms (CV) at different scan rates (from 50 mV/s to 200 mV/s). (a) Ni_3_S_2_@V-NiFe(III) LDH/NF, (b) V-Fe(OH)_3_/NF, (c) Ni_3_S_2_@NiFe(III) LDH/NF and (d) Ni_3_S_2_/NF; Figure S3. ECSA-normalized OER polarization curves with *iR* correction; Figure S4. Bode plots of (a) Ni_3_S_2_@V-NiFe(III) LDH/NF, (b) V-Fe(OH)_3_/NF, (c) Ni_3_S_2_@NiFe(III) LDH/NF and (d) Ni_3_S_2_/NF at potential range from 1.46 to 1.54 V vs RHE; Figure S5. The 20th cycle CV curves without *iR* correction of Ni_3_S_2_@V-NiFe(III) LDH/NF, V-Fe(OH)_3_/NF, Ni_3_S_2_@NiFe(III) LDH/NF, Ni_3_S_2_/NF and NF; Table S1. Comparison of the OER performance of Ni_3_S_2_@V-NiFe(III) LDH/NF catalyst; Table S2. Comparison of reduction peak potentials of the 1st cycle CV curves about Ni_3_S_2_@V-NiFe(III) LDH/NF, Ni_3_S_2_@NiFe(III) LDH/NF, V-Fe(OH)_3_/NF and Ni_3_S_2_/NF.
