# Supplementary material for: Construction of Heterostructured Ni3S2@V-NiFe(III) LDH for Enhanced OER Performance
Source: Molecules. 2024 Dec 20;29(24):6018. doi: 10.3390/molecules29246018 (PMC11676444; doi:10.3390/molecules29246018)
Supplement: Supplementary file 1 [file molecules-29-06018-s001.zip › molecules-3358188-supplementary.pdf]

Construction of Heterostructured Ni_3S_2@V-NiFe(III) LDH for Enhanced OER Performance

Qianqian Dong [1], Qijun Zhong [1], Jie Zhou [1], Yuhao Li [1], Yujing Wang [1], Jiayang Cai [2], Shuangwei Yu [1], Xiong He [1,*] and Shaohui Zhang [1]

[1] Liuzhou Key Laboratory of New Energy Vehicle Power Lithium Battery, Guangxi Engineering Research Center for Characteristic Metallic Powder Materials, School of Electronic Engineering, Guangxi University of Science and Technology, Liuzhou 545000, China; isdongqq@163.com (Q.D.); zqj17878906682@163.com (Q.Z.); 17586600924@163.com (J.Z.); q2476166290@126.com (Y.L.); wang_yu_jing01@163.com (Y.W.); yushuangwei2023@163.com (S.Y.); zhangshaohui@gxust.edu.cn (S.Z.)

[2] Guangxi Key Laboratory of Green Processing of Sugar Resources, College of Biological and Chemical Engineering, Guangxi University of Science and Technology, Liuzhou 545006, China; cjy193677464@163.com

* Correspondence: hexiong@gxust.edu.cn

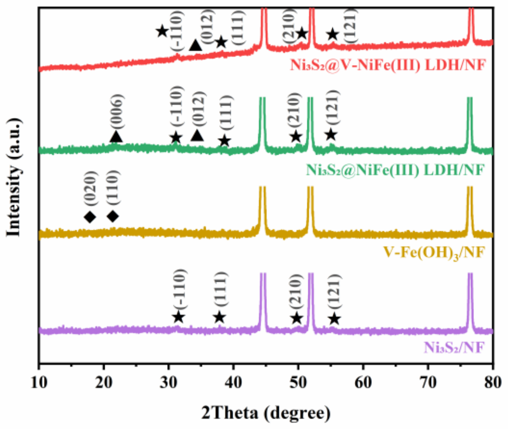

Figure S1. XRD patterns of all the samples.

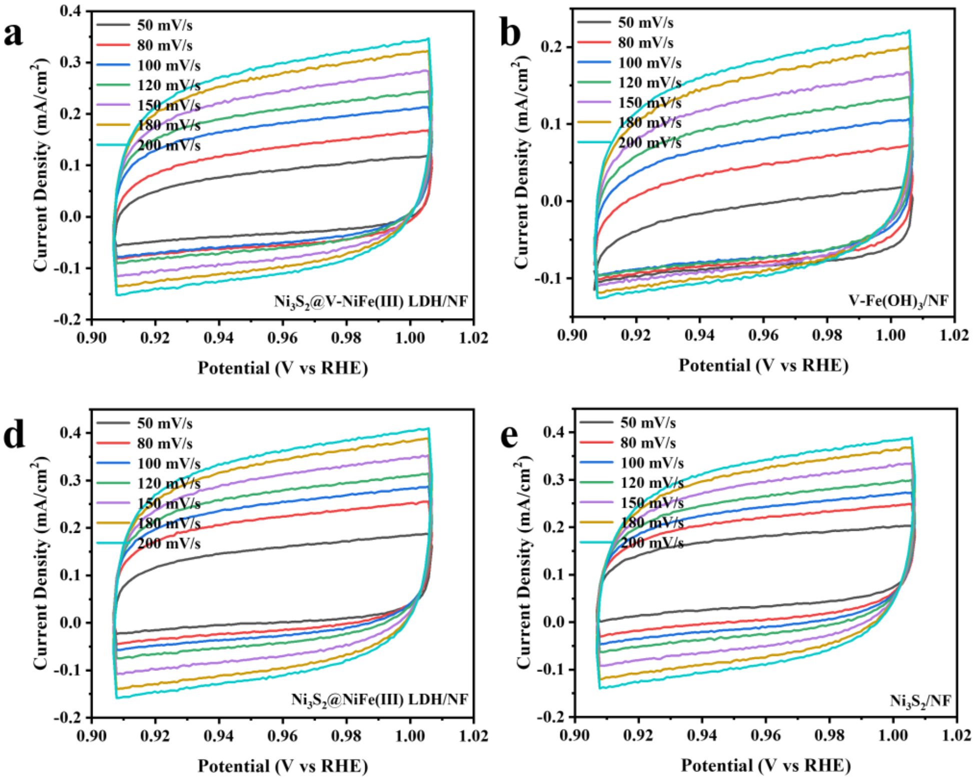

Figure S2. Cyclic voltammograms (CV) at different scan rates (from 50 mV/s to 200 mV/s). (a) Ni_3S_2@V-NiFe(III) LDH/NF, (b) V-Fe$(OH)_3$/NF, (c) Ni_3S_2@NiFe(III) LDH/NF and (d) Ni_3S_2/NF.

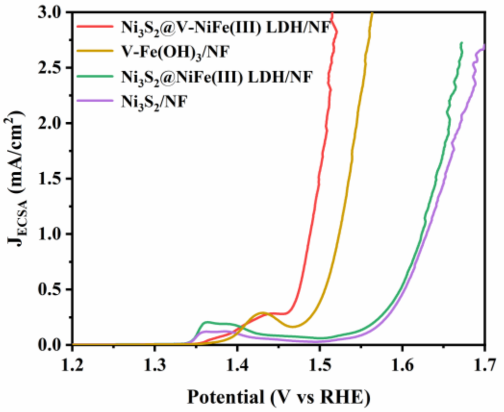

Figure S3. ECSA-normalized OER polarization curves with *iR* correction.

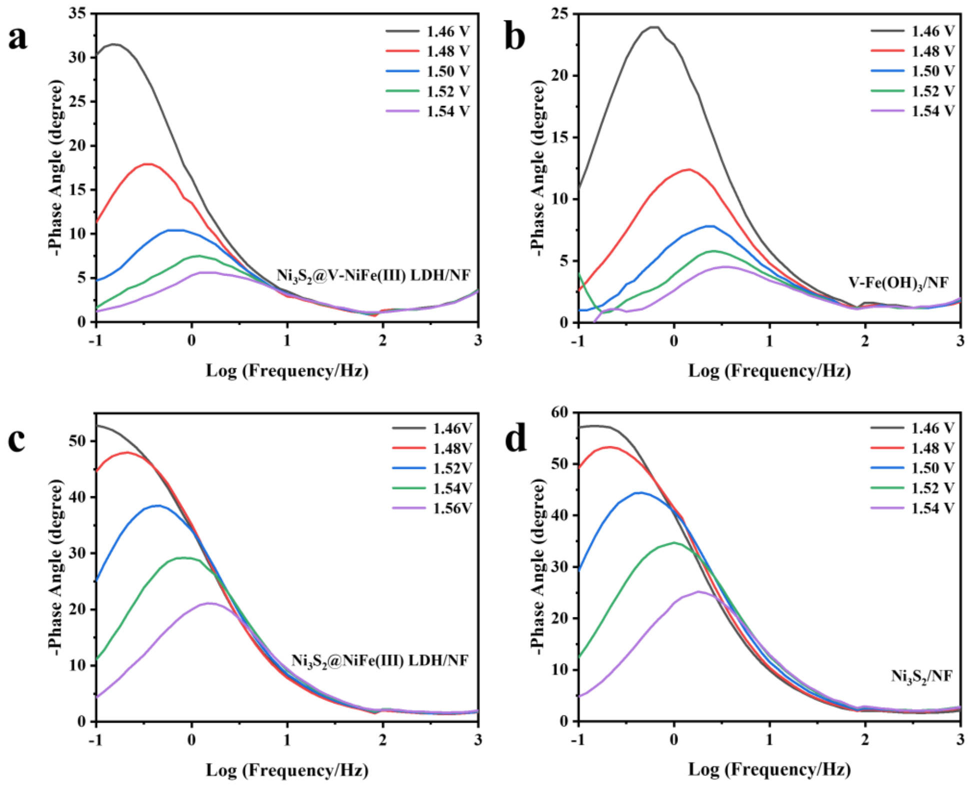

Figure S4. Bode plots of (a) Ni_3S_2@V-NiFe(III) LDH/NF, (b) V-Fe$(OH)_3$/NF, (c) Ni_3S_2@NiFe(III) LDH/NF and (d) Ni_3S_2/NF at potential range from 1.46 to 1.54 V vs RHE.

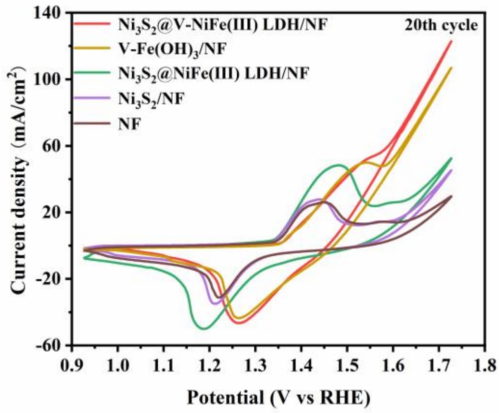

Figure S5. The 20th cycle CV curves without *iR* correction of Ni_3S_2@V-NiFe(III) LDH/NF, V-Fe$(OH)_3$/NF, Ni_3S_2@NiFe(III) LDH/NF, Ni_3S_2/NF and NF.

Table S1. Comparison of the OER performance of Ni_3S_2@V-NiFe(II) LDH/NF catalyst.

Electrocatalysts	*J* mA/cm^2	η (mV)	Tafel slope (mV/dec)	Stability	Reference
**Ni_3S_2@V-NiFe(III) LDH/NF**	**100<br>50<br>20**	**280<br>259<br>241**	**45.4**	**100 h at 50 mA/cm^2**	**This work**
VSNC	10<br>50	290<br>340	68	50 h at 10 mA/cm^2	Adv. Funct. Mater. 2023, 33, 2211530
NiFe LDH-TEA	10	261	32.5	20 h at 15 mA/cm^2	Journal of Colloid and Interface Science 629 (2023) 610–619
NiFe-$V_{1.0}$	10	255	43.5	20 h at 1.58 V vs. RHE	Journal of Alloys and Compounds 885 (2021) 160929
Ta-NiFe LDH	50	260	58.95	20 h at 100 mA/cm^2	Chemical Engineering Journal 403 (2021) 126297
V-Ni_3S_2@NiFe LDH	100	286	32.5	24 h at 10,100 mA/cm^2	J. Mater. Chem. A, 2019,7, 18118-18125
CoFeV-0.25/NF	100<br>50	330<br>290	57	30 h at 50 mA/cm^2	ACS Sustainable Chem. Eng. 2019, 7, 16828−16834
$NiFe_2O_4$-NiOOH	30	240	55	100 h at ~80 mA/cm^2	Sci China Mater. 2017, 60(4): 324–334
VOOH	10	270	68	24 h at 10 mA/cm^2	Angew. Chem. Int. Ed. 2017, 56, 573 – 577

Note: VSNC is V_2(Sn, Ni)C MAX phase catalysts. NiFe LDH-TEA is triethanolamine-intercalated NiFe-layered double hydroxides. NF is Ni foam.

Table S2. Comparison of reduction peak potentials of the 1st cycle CV curves about Ni_3S_2@V-NiFe(III) LDH/NF, Ni_3S_2@NiFe(III) LDH/NF, V-$Fe(OH)_3$/NF and Ni_3S_2/NF.

Electrocatalysts	Potential of Reduction peak (V vs RHE)		
	1st CV	20th CV	ΔE
Ni_3S_2@V-NiFe(III) LDH/NF	1.340	1.264	-0.076
V-$Fe(OH)_3$/NF	1.362	1.264	-0.098
Ni_3S_2@NiFe(III) LDH/NF	1.227	1.188	-0.039
Ni_3S_2/NF	1.215	1.212	-0.003
NF	1.216	1.221	0.005
